# Trion X^+^ in vertically coupled type II quantum dots in threading magnetic field

**DOI:** 10.1186/1556-276X-7-532

**Published:** 2012-09-26

**Authors:** Sindi Horta-Piñeres, Gene Elizabeth Escorcia-Salas, Ilia D Mikhailov, José Sierra-Ortega

**Affiliations:** 1 Group of Investigation in Condensed Matter Theory, Universidad del Magdalena, Santa Marta, AA 731, Colombia; 2 Universidad de Sucre, Sincelejo, AA 406, Colombia; 3 Universidad Industrial de Santander, Bucaramanga, AA 678, Colombia

**Keywords:** Quantum dots, Adiabatic approximation, Trion, 78.67.-n, 71.35.Pq, 73.21.La

## Abstract

We analyze the energy spectrum of a positively charged exciton confined in a semiconductor heterostructure formed by two vertically coupled, axially symmetrical type II quantum dots located close to each other. The electron in the structure is mainly located inside the dots, while the holes generally move in the exterior region close to the symmetry axis. The solutions of the Schrödinger equation are obtained by a variational separation of variables in the adiabatic limit. Numerical results are shown for bonding and anti-bonding lowest-lying of the trion states corresponding to the different quantum dots morphologies, dimensions, separation between them, thicknesses of the wetting layers, and the magnetic field strength.

## Background

During the last few years, there has been much interest in the study of quantum dots (QDs), which are structures in which charge carriers are confined in all three dimensions. The quantum dots have opened the possibility to fabricate both artificial atoms and molecules with novel and fascinating optoelectronic properties which are not accessible in bulk semiconductor materials. Especially, the self-assembled QDs are considered to be very promising for possible applications, such as QD lasers, due to their large confinement energy and high optical quality. An attractive route for nanostructuring semiconductor materials is offered by the self-assembled quantum dots (SAQDs) which are formed via the Stranski-Krastanow growth mode by depositing a material on a substrate with a different lattice parameter [1-5]. The analogy with atoms can be strengthened by the capture of carriers with opposite charges. In spite of the fact that SAQDs can possess different morphologies such as disks, cones, rings, and lenses [1], they are, in general, thin layers and have, for the most part, a small height-to-base aspect ratio whose typical values vary between 0.029 and 0.067 [3]. The motion of the carriers in such structures is quasi-two-dimensional but due to the tunneling of the carriers through finite height barrier along the crystal growth direction, they can escape outside of the layer, with a considerable probability. Besides, if the probabilities of the electron and the hole tunneling are different, the opposite charge density in the central part inside the SAQD and the peripheral regions around SAQDs can be induced by a captured electron-hole pair. It is established that the quantum confinement of few particles systems, e.g., neutral and negatively charged donors (D^0^ and D^−^, respectively), excitons (X), trions (X^−^, X^+^) and biexcitons, enhances the binding energy allowing their observation even at the room temperature. The energy spectra of these systems governed by the interplay between tunneling, confinement, and correlation effects have been studied by using different methods, such as variational [6], diagonalization [7-9], and finite elements [10]. Although these techniques give consistent results with the experimental data, they entail a tedious computational work. Besides, the great majority of the analyzed models are two-dimensional, and they left to aside the effects related to the disk height. Recently, in order to calculate ground state energies of D^0^, D^−^, and X in the semiconductor heterostructures, it has been proposed as a simple variational procedure [11-13] related to fractal dimension scheme [14]. In this paper, we analyze the energy spectrum of a positively charged exciton confined in a semiconductor heterostructure formed by two vertically coupled, axially symmetrical type II quantum dots located close to each other. The electron in the structure is mainly located inside the dots, while the holes generally are placed in the exterior region close to the symmetry axis. The solutions of the Schrödinger equation are obtained by a variational separation of variables in the adiabatic limit. Numerical results are shown for bonding and anti-bonding lowest-lying of the trion states corresponding to different quantum dots morphologies, dimensions, the separation between them, thicknesses of the wetting layers, and the magnetic field strength.

## Methods

We assume that the adiabatic approximation can be used as a starting point for a qualitative analysis of the ground state energy of X^+^, taking into account that the effective hole mass in the In_0.55_Al_0.45_As material is essentially greater than the effective mass of the electron, and this circumstance is more relevant in conditions of strong confinement. In this approximation the Hamiltonian of the X^+^ can be expressed in terms of the Hamiltonian of the D_2_^+^. To analyze the spectrum of the X^+^ within framework of the adiabatic approximation, we first consider this complex under condition when the holes position vectors **r**_*h*1_ and **r**_*h*2_ are fixed, and this structure can be considered as a donor complex D_2_^+^. Being **r**_*e*_ as the position vector of the electron and **r**_*h*1_, **r**_*h*2_ as the position vectors of the ions, the Hamiltonian for the donor D_2_^+^ ground state in a structure with the confinement potential *V*_*e*_(**r**_*e*_)due the structural non-homogeneity and in the presence of the external magnetic field *B* applied along the symmetry axis can be written as:

(1) $$\begin{array}{l} H_{{{\text{D}}_{2}}^{+}}\left(r_{e},r_{h1},r_{h2}\right)=H_{0e}\left(r_{e}\right)-2/\left|r_{e}-r_{h1}\right|-2/\left|r_{e}-r_{h2}\right|;\\ H_{0}\left(r_{e}\right)=-\eta_{e}\left[\Delta_{e}+i\gamma \frac{\partial }{\partial \vartheta_{e}}+\frac{\gamma^{2}\;\rho_{e}^{2}}{4}\right]+V_{e}\left(r_{e}\right) \end{array}$$

The effective Bohr radius $a_{0}^{*}=\hbar^{2}\varepsilon /\mu \;e^{2}$ as the unit of length, the effective Rydberg $\text{Ry}*=e^{2}/2\varepsilon \;a_{0}^{*}=\hbar^{2}/2\mu \;{a_{0}^{*}}^{2}$ as the energy unit, and γ=eℏB/2μcRy* as the unit of the magnetic field strength have been used in Hamiltonian (Equation 1), $m_{e}^{*}$ and $m_{h}^{*}$ the electron and the hole effective masses and $\mu =m_{e}^{*}m_{h}^{*}/\left(m_{e}^{*}+m_{h}^{*}\right)$ as the reduced mass. The following notations have been used: $\eta_{k}=m_{k}^{*}/\mu$ and the cylindrical coordinates **r**_*k*_=(*ψ*_*k*_, *ϑ*_*k*_, *z*_*k*_) labeled by *k* = *h*1, *h*2, *e* corresponding to the first hole, the second hole, and the electron, respectively. For the sake of simplicity, we consider a model of the QD with infinite-barrier confinement potential, *V*_*e*_(*ρ*_*e*_, *z*_*e*_) = 0, inside the QD and to infinity otherwise, which restricts the electron only inside the QD, and for the holes located away from QDs, *V*_*h*_(*ρ*_*h*_, *z*_*h*_) = 0, otside the QD and to infinity inside them. The eigenfunctions *φ*_*e*_(**r**_*e*_) and eigenvalues $E_{{{\text{D}}_{2}}^{+}}\left(r_{h1},r_{h2}\right)$ of the Hamiltonian (Equation 1) solutions of the wave equation

(2) $$H_{{{\text{D}}_{2}}^{+}}\left(r_{e},r_{h1},r_{h2}\right)\varphi_{e}\left(r_{e}\right)=E_{{{\text{D}}_{2}}^{+}}\left(r_{h1},r_{h2}\right)\varphi_{e}\left(r_{e}\right)$$

can be found by using the method described in the previous paper [15]. The positively charged exciton X^+^ Hamiltonian can be written in terms of the D_2_^+^ Hamiltonian (Equation 1) as follows:

(3) $$\begin{array}{ll} \;H_{X^{+}}\left(r_{e},r_{h1},r_{h2}\right) & =H_{{{\text{D}}_{2}}^{+}}\left(r_{e},r_{h1},r_{h2}\right)+H_{0}\left(r_{h1}\right)\\ & \;+H_{0}\left(r_{h2}\right)+2/\left|r_{h1}-r_{h2}\right|;\\ \;H_{0}\left(r_{k}\right)=-\eta_{h}\left[\Delta_{k}\right) & \left(+i\gamma \frac{\partial }{\partial \vartheta_{k}}+\frac{\gamma^{2}\;\rho_{k}^{2}}{4}\right]+V_{h}\left(r_{k}\right);\;k=h1,h2. \end{array}$$

Following the adiabatic approximation standard procedure, we seek the eigenfunction of the Hamiltonian (Equation 3) as $\Psi_{X^{+}}\left(r_{e},r_{h1},r_{h2}\right)=\varphi_{e}\left(r_{e},r_{h1},r_{h2}\right)\psi \left(r_{h1},r_{h2}\right)$, where *ψ*(**r**_*h*1_, **r**_*h*2_) is the part of the wave function describing a slow holes' motion which is a solution of the following wave equation:

(4) $$\begin{array}{c} -\eta_{h}\Delta_{h1}\psi -\eta_{h}\Delta_{h2}\psi \\ +\left\{E_{{{\text{D}}_{2}}^{+}}\left(r_{h1},r_{h2}\right)+V_{h}\left(r_{h1}\right)+V_{h}\left(r_{h2}\right)+2/\left|r_{h1}-r_{h2}\right|\right\}\\ \psi =E_{X^{+}}\psi \end{array}$$

Here, $E_{{{\text{D}}_{2}}^{+}}\left(r_{h1},r_{h2}\right)$, the energy of the fast electron motion as a function of the holes positions, presents potential curves for the holes' slow motion.

Once the energies $E_{{{\text{D}}_{2}}^{+}}\left(r_{h1},r_{h2}\right)$ for the different electron levels as functions of the holes positions are found, then the wave in Equation 4 can be solved approximately by using the method of symmetry structures [16] in which the problem in Equation 3 is reduced to the following one-particle wave equation:

(5) $$-\eta_{h}\Delta_{h}\psi +\left\{E_{{{\text{D}}_{2}}^{+}}\left(r_{h},-r_{h}\right)+V_{h}\left(r_{h}\right)+1/\left|r_{h}\right|\right\}\psi =\left(E_{X^{+}}/2\right)\psi \text{.}$$

**Figure 1:**
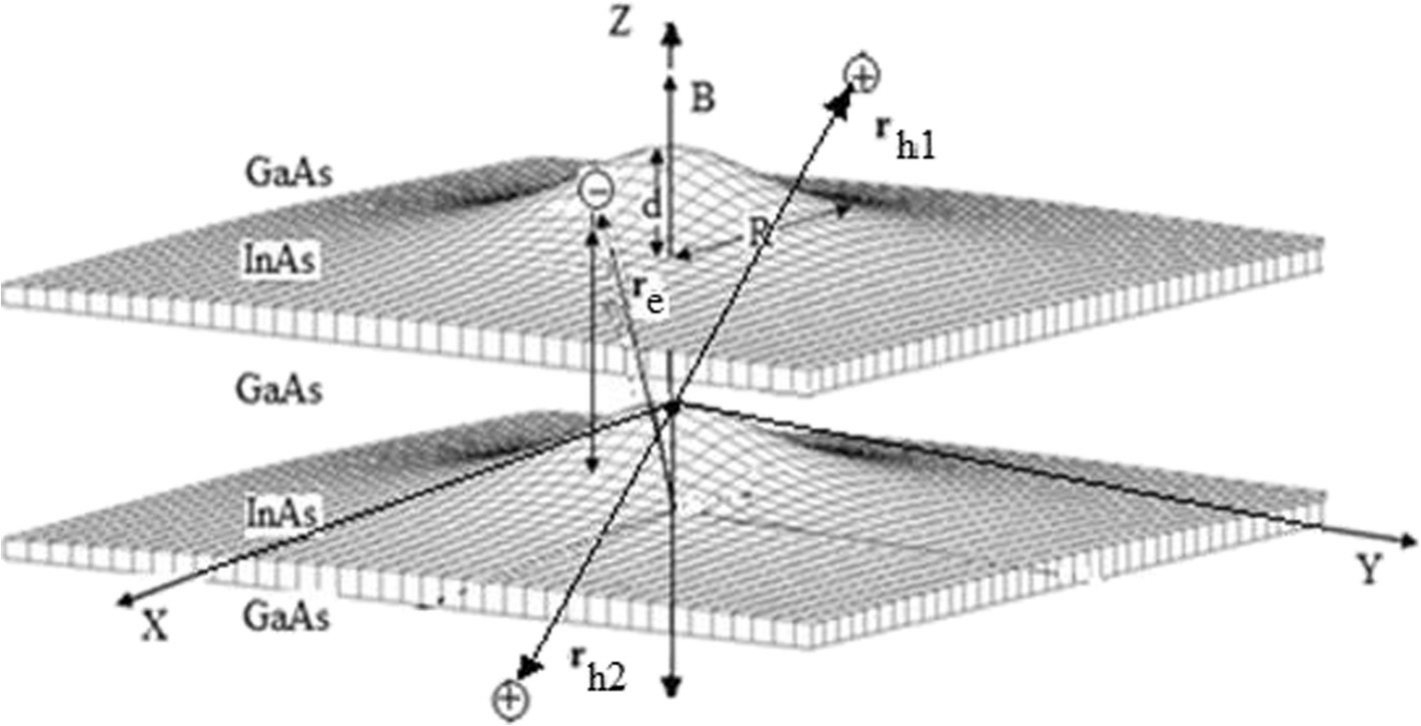
**Scheme of a trion X**^**+ **^**confined in a type II vertically coupled quantum dots.**

Schematically, the structure corresponding to Equation 5 is presented in Figure 1. The different sublevels of the X^+^ corresponding to the different energies of the hole motion in *Z* direction can be analyzed by solving the one-dimensional wave equation (Equation 5). In our calculations we use to aim the trigonometric sweep method [17].

## Results and discussion

**Figure 2:**
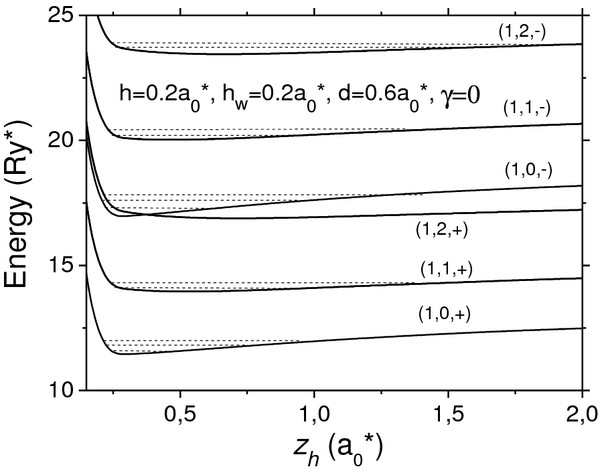
**Potential curves**$E_{{{\text{D}}_{2}}^{+}}\left(d/2+z_{h},-d/2-z_{h}\right)$**for the electron confined in vertically coupled lens-shaped QDs.** Solid lines represent energy levels of the Trion X^+^ and dashed lines, Trion X^+^. With separation, *d,* between layers and distance, *Z*_*h*_, between the hole and the layer for zero-magnetic field case (*γ* = 0).

In Figure 2 we present the energies of some lower electron levels as functions of the distance *z*_*h*_ between the hole and the adjacent semiconductor layer in the framework of the symmetrical structure model for the case when the height of the lens-shaped QD over the wetting layer of *h*_*w*_ = 0.2*a*_0_* thickness is also equal to *h* = 0.2*a*_0_*, while the separation between layers is *d* = 0.6*a*_0_*.

The corresponding potential curves are indicated by two-electron quantum numbers (*n*_*e*_, *l*_*e*_) and by symbols (+) and (−) for the bonding and anti-bonding levels, respectively. It is seen that the electron energies corresponding to different levels initially descends as the distance between holes and the layers *Z*_*h*_ decreases, while the electrostatic attraction between electron and holes is greater than the repulsion from the layer provided by the structural confinement. As *Z*_*h*_ further decreases, the repulsion from the layer provided by the increasing confinement becomes significant and the potential energy becomes to climb up sharply. Also, in Figure 2 we show the holes' vibrational sublevels (dashed lines) with gaps significantly smaller than the corresponding values between electronic levels (solid lines). Similarly, the gaps between bonding and anti-bonding levels with the same quantum numbers, (*n*_*e*_, *l*_*e*_), are larger than the gaps between the two levels with the same parity and different quantum numbers. Additionally, one can see that energies corresponding to the bonding states are generally inferior, and there is only one crossover between the lower anti-bonding potential curve and the upper bonding one. The parameter *Z*_*h*_ tends to infinitize the energies of the potential curves approach asymptotically to electronic energy levels, while the dash lines in Figure 2 show energy levels (ground and excited) of the positively charged trion.

**Figure 3:**
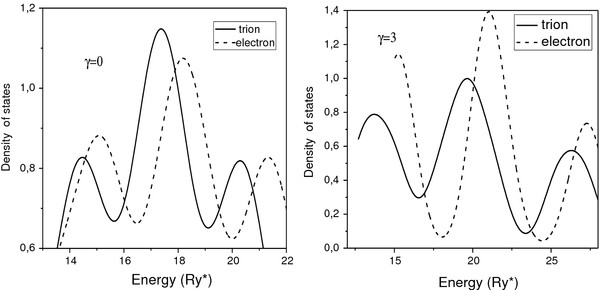
**Density of states for trion and electron in vertically coupled lens-shaped QDs.** Density of states for trion (solid lines) and electron (dashed lines) in vertically coupled lens-shaped QDs for (left image), (*γ* = 0) and (right image), (*γ* = 3).

In Figure 3 we present the density of states (DOS) corresponding to the electron (dash lines) and to the positively charged trion (solid lines) for cases of the zero magnetic field (*γ* = 0) and (*γ* = 3). In both cases one can observe the presence of three separated peaks which we associate to the bonding, mixed, and anti-bonding states, respectively. The first peak is a superposition of the lines corresponding to the lower bonding states, the intermediate peak is related to a superposition of the upper bonding and the lower anti-bonding states, and the last peak is due to a superposition of the upper anti-bonding states.

When comparing the curves of DOS for the trion and electron, one can see that the additional attraction between the electron and holes in trion provides a displacement of all the peaks toward the region with lower energies. On the other hand, an external magnetic field enhances the difference between the curves of DOS for the electron and the trion due to the fact that the splitting between energy levels with positive and negative angular momentum states for the trion is larger than for the electron. Therefore, it is seen in Figure 3 a more significant broadening of the peaks for the trion in the presence of the magnetic field (*γ* = 3). Also in Figure 3, one can observe a slight displacement of the peaks provided by the magnetic field toward the region of higher energies due to the diamagnetic term $\gamma^{2}\;\rho^{2}/4$ in the Hamiltonian.

**Figure 4:**
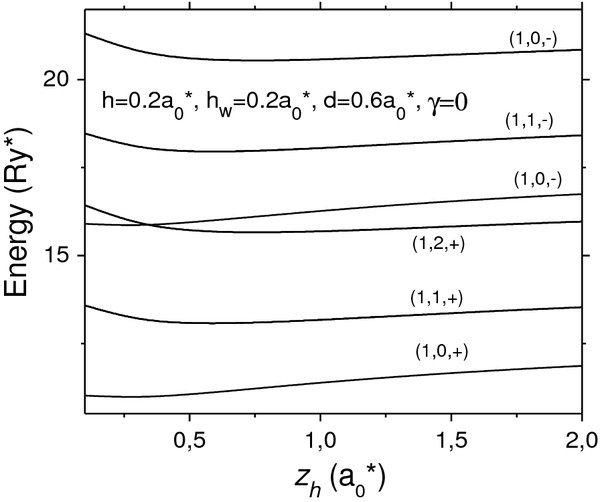
**The same as in Figure**2** but for disk-shaped QDs.**

**Figure 5:**
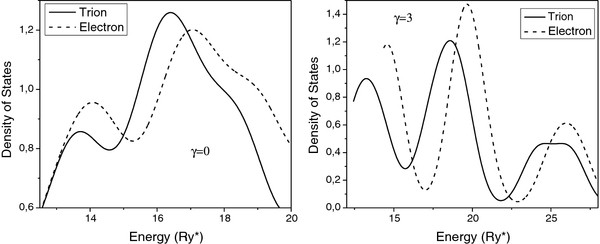
**The same as in Figure**3** but for disk-shaped QDs.**

In Figures 4 and 5 we display the similar results for vertically coupled disk-shaped QDs. It is seen that the potential curves for the disks are analogous to those for the lens in Figure 2 with a single but very important difference: the electron energies and gaps between them in the lens and in Figure 4 are lower than the correspondent values in the lens in Figure 2. It is due to the fact that the in-plane confinement in lens is stronger than one in disk resulting in a weak resolution of the three peaks in the zero-magnetic field case (*γ* = 0) as it is seen in Figure 5, where the third peak is transformed in a right-side shoulder at DOS curve.

However, as the magnetic field is sufficiently strong, the electron becomes more strongly confined within a region about the axis and the energy spectrum becomes analogous to one in lens. In consequence, the DOS in disk-shaped vertically coupled QDs for the magnetic field in Figure 5 (*γ* = 3) becomes similar to the lens-shaped QDs in Figure 3.

## Conclusions

In short we propose a simple numerical procedure for calculating the energy spectrum of a positively charged exciton confined in a semiconductor heterostructure that are formed by two vertically coupled, axially symmetrical type II quantum dots located close to each other. The electron in the structure is mainly located inside the dots, while the holes generally are placed in the exterior region close to the symmetry axis. Our calculation includes some important characteristic of the heterostructure such as the possibility of the variation of the QD morphology. We found that the holes' vibrational sublevels with gaps significantly smaller than the corresponding values between the electronic levels and the gaps between bonding and anti-bonding levels with the same quantum numbers are larger than the gaps between two levels with the same parity and different quantum numbers. Also we found that the splitting between energy levels with positive and negative angular momentum states for the trion is larger than for the electron, and the external magnetic field enhances the difference between curves of DOS for the electron and the trion.

## Competing interests

The authors declare that they have no competing interests.

## Authors’ contributions

All authors contributed equally to this work. JSO created the analytic model with contributions from IM, SHP and GES performed numerical calculations and wrote the manuscript. All authors discussed the results and implications and commented on the manuscript at all stages. All authors read and approved the final manuscript.
